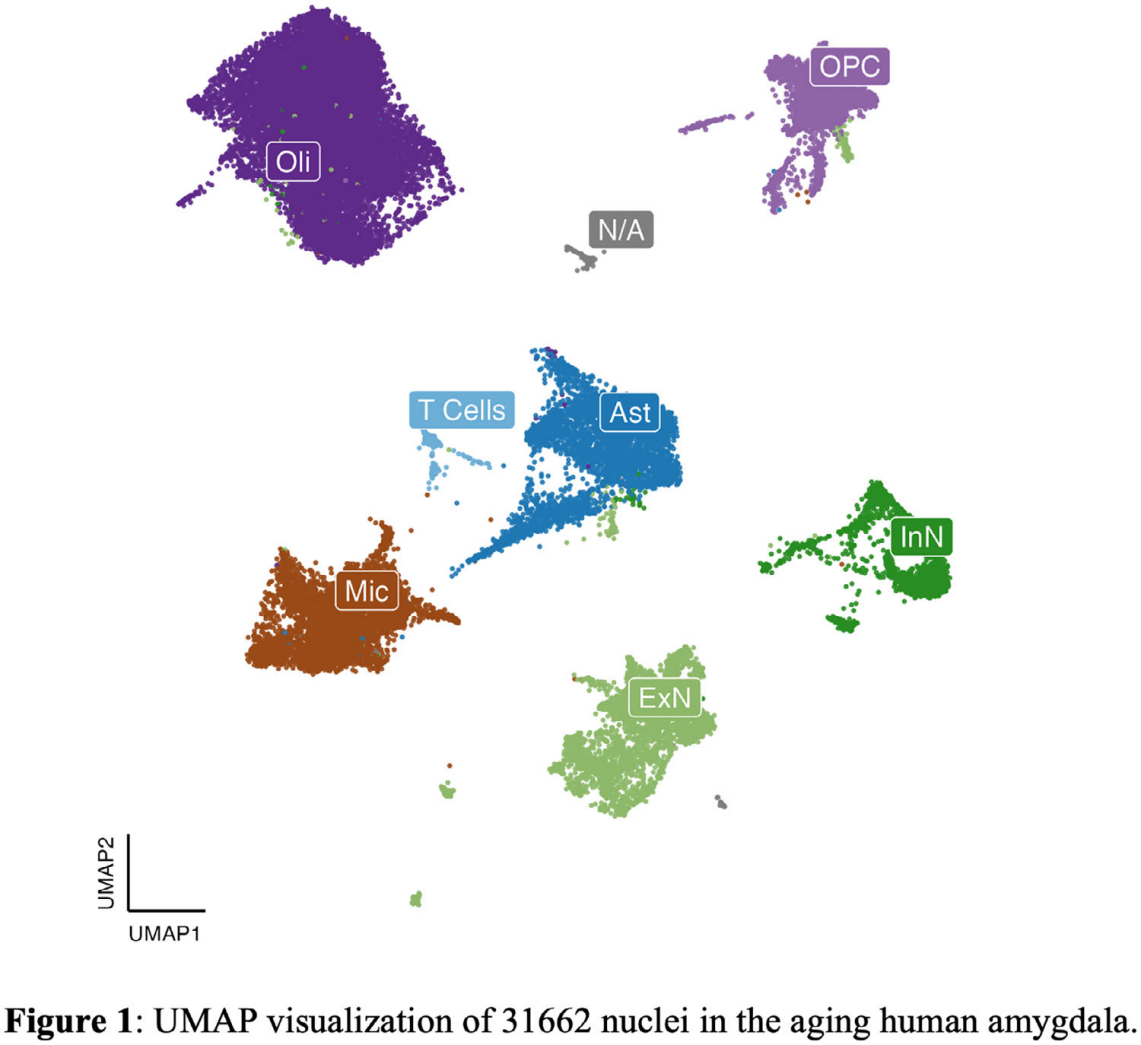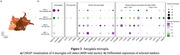# A single‐cell atlas of the aging human amygdala: a pilot study

**DOI:** 10.1002/alz70855_104999

**Published:** 2025-12-23

**Authors:** Zheng Yin, Ling Teng, Ya Zhang, David W Gagliardi, Ryan Johnson, David H. Adamowicz, Jijing Wang, Lei Liu, Jasmeer P. Chhatwal, Philip L. De Jager, David A. A. Bennett, Julie A Schneider, Vladislav A Petyuk, Vilas Menon, Hyun‐Sik Yang

**Affiliations:** ^1^ Mass General Brigham, Boston, MA, USA; ^2^ Harvard Medical School, Boston, MA, USA; ^3^ The Broad Institute of MIT and Harvard, Cambridge, MA, USA; ^4^ Center for Translational & Computational Neuroimmunology, Columbia University Irving Medical Center, New York, NY, USA; ^5^ Rush University Medical Center, Chicago, IL, USA; ^6^ Columbia University Irving Medical Center, New York, NY, USA; ^7^ Pacific Northwest National Laboratory, Richland, WA, USA

## Abstract

**Background:**

The amygdala is involved in early stages of multiple neurodegenerative disorders such as Alzheimer's disease (AD), limbic‐predominant age‐related TDP‐43 encephalopathy neuropathologic change (LATE‐NC), and Lewy body disease (LBD). However, the single‐cell architecture of the aging human amygdala is not well characterized.

**Method:**

The basolateral amygdala was dissected from 9 brain donors recruited at Rush Alzheimer's Disease Center. We generated 10x single‐nucleus multi‐ome (RNA + Assay for Transposase‐Accessible Chromatin [ATAC]‐seq) data: we pooled 3 samples per batch and then demultiplexed them using freemuxlet. The following QC parameters were used: mitochondrial RNA content<10%, RNA>1000 UMIs, and ATAC>100 UMIs. We used Harmony and Weighted Nearest Neighbor (WNN) algorithms to integrate multi‐omic data across batches. We identified major brain cell types, cell states, and their marker genes, and visualized the clusters using uniform manifold approximation and projection (UMAP).

**Result:**

All 9 participants (6 females; average age of death 89.7±4.95) had at least some AD pathology, 6 had LATE‐NC, and 2 had LBD pathology. Our pipeline yielded 31,662 high‐quality single‐nucleus multi‐omes. We observed well‐defined clusters of astrocytes (Ast), excitatory (ExN) and inhibitory (InN) neurons, microglia (Mic), oligodendrocytes (Oli), oligodendrocyte progenitor cells (OPC), and T cells. We identified 3 Ast, 7 ExN, 4 InN, 4 Mic, 5 Oli, 3 OPC, and 1 T cell states. In our focused analyses of Mic, APOE was highly expressed in two abundant Mic states, one of which also highly expressed TREM2; markers of human AD‐associated microglia states (Green et al., Nature 2024) and mouse disease‐associated microglia (DAM) (Keren‐Shaul et al., Cell 2017) were also highly expressed in those two states. Notably, the observed proportion of these states (73.9±11.2% of total microglia) were much higher than that from the frontal cortex of AD patients (10.9±9.0%). In addition, we observed a homeostatic‐like state (high P2RY12, CX3CR1) and a state highly expressing interferon (IFN) response genes (IFIT2, IFIT3).

**Conclusion:**

Our pilot study demonstrated the feasibility of generating high‐quality, large‐scale single‐cell multi‐omic data from the human amygdala. We observed abundant AD‐associated microglia in amygdala, suggesting the possibility that the regional vulnerability of amygdala to multiple neurodegenerative disorders might be in part due to different glial composition.